# Systematic literature review of instruments that measure the healthfulness of food and beverages sold in informal food outlets

**DOI:** 10.1186/s12966-022-01320-1

**Published:** 2022-07-16

**Authors:** Catalina Medina, Maricela Piña-Pozas, Tania C. Aburto, Julissa Chavira, Uzzi López, Mildred Moreno, Armando G. Olvera, Citlali Gonzalez, Terry T-K Huang, Simón Barquera

**Affiliations:** 1 Center for Health and Nutrition Research, National Institute of Public Health, Mexico. Avenida Universidad 655, Santa María Ahuacatitlán. CP. 06210, Cuernavaca, Morelos Mexico; 2 Center for Information for Public Health Decisions, National Institute of Public Health, Mexico. Avenida Universidad 655, Santa María Ahuacatitlán. CP. 06210, Cuernavaca, Morelos Mexico; 3 School of Engineering and Architecture (ESIA), National Polytechnic Institute (IPN), México, Avenida Fuentes de los Leones 28, Lomas de Tecamachalco. CP. 53955. Tecamachalco, Naucalpan, Mexico; 4 Center for Systems and Community Design and NYU-CUNY Prevention Research Center, Graduate School of Public Health and Health Policy, City University of New York, 55W. 125 Street, Room 803, New York, NY 10027 USA

**Keywords:** Food environment, Measurements, Informal food outlets, Literature review, Street food

## Abstract

**Background:**

Informal food outlets, defined as vendors who rarely have access to water and toilets, much less shelter and electricity, are a common component of the food environment, particularly in many non-Western countries. The purpose of this study was to review available instruments that measure the quality and particularly the healthfulness of food and beverages sold within informal food outlets.

**Methods:**

PubMed, LILACS, Web of Science, and Scopus databases were used. Articles were included if they reported instruments that measured the availability or type of healthy and unhealthy foods and beverages by informal food outlets, were written in English or Spanish, and published between January 1, 2010, and July 31, 2020. Two trained researchers reviewed the title, abstract and full text of selected articles; discrepancies were solved by two independent researchers. In addition, the list of references for selected articles was reviewed for any additional articles of relevance. The quality of published articles and documents was evaluated using JBI Critical appraisal checklist for analytical cross-sectional studies.

**Results:**

We identified 1078 articles of which 14 were included after applying the selection criteria. Three additional articles were considered after reviewing the references from the selected articles. From the final 17 articles, 13 measurement tools were identified. Most of the instruments were used in low- and middle-income countries (LMIC). Products were classified as healthy/unhealthy or produce/non-produce or processed/unprocessed based on availability and type. Six studies reported psychometric tests, whereas one was tested within the informal food sector.

**Conclusions:**

Few instruments can measure the healthfulness of food and beverages sold in informal food outlets, of which the most valid and reliable have been used to measure formal food outlets as well. Therefore, it is necessary to develop an instrument that manages to measure, specifically, the elements available within an informal one. These actions are extremely important to better understand the food environment that is a central contributor to poor diets that are increasingly associated with the obesity and Non-communicable disease (NCD) pandemic.

**Supplementary Information:**

The online version contains supplementary material available at 10.1186/s12966-022-01320-1.

## Introduction

The prevalence of nutrition-related non-communicable diseases (NCDs) including obesity, type 2 diabetes, hypertension, and cardiovascular disease, has increased globally, particularly in low- and middle-income countries (LMICs) [1, 2]. Moreover, most LMICs currently face the double burden of malnutrition, with a prevalence of stunting among children 0–59 months of 29.1% [3, 4].

The food environment is described as the availability, affordability, convenience, and desirability of various foods, [5] and has been a focus of increasing research interest. Food environments are hypothesized to partially explain the increase in the prevalence of obesity by providing greater access to unhealthy foods and/or lower access to healthy ones [6]. Several systematic reviews have been published summarizing the evidence on the impact of food environments on the association with nutrition- and health-related outcomes, as well as for methodologies used to measure food environments [7–12]. However, most of this research has been conducted in high-income countries, where informal food outlets or street food might not be as prevalent as in LMICs [13].

Informal food outlets are defined as vendors who rarely have access to water, toilets, shelter and electricity [14]. Informal food outlets could include street food vending, mobile food outlets, and open-air markets [15–17]. According to a systematic review, daily energy intake from foods consumed from informal food outlets ranged from 13 to 50% in adults, and from 13 to 40% in children in LMICs [18]. This study also documented the wide variety of street foods offered by informal food outlets, including healthy items such as fresh fruits, vegetables, and cooked legumes, but also unhealthy items such as soft drinks, cookies, pastries, deep-fried fish and meats, deep-fried snacks, along with other ultra-processed products. The range of foods offered also spans different processing levels, from minimally processed foods (e.g., fresh fruits), prepared dishes - either in advance or at the moment of purchase (e.g., stews and deep-fried fish), to ultra-processed foods (e.g., soft drinks and candies) [18].

In recent years, there has been an increase in the prevalence of away-from-home eating around the world and within LMICs [19–25]. Eating away from home has been associated with a high intake of low-quality foods, high in critical nutrients including saturated fat, cholesterol, and sodium [26]. Recently, there is a growing tendency of the food industry to blame street food on the high availability of unhealthy traditional food [27, 28]. However, even though several studies have assessed the nutrient contribution of street foods to dietary intake, [18, 29] evidence about the dietary quality of street food is limited. This might be partly explained by the wide variety of foods offered by informal food vendors and consequently, the complexity of measuring and standardizing such food environments. However, given the fundamental role of dietary intake in the double burden of malnutrition and the potential contribution of informal food options to dietary intake around the world, there is an urgent need for a standardized instrument to assess the dietary quality of street food to characterize it, explore associations with nutrition and health outcomes, and allow comparisons between places and across time. Therefore, the present study aimed to review the available instruments that measured the healthfulness of food and beverages sold within informal food outlets.

## Methodology

### Informal food outlets

Informal food outlets are defined as vendors who rarely have access to water, toilets, shelter and electricity [14]. Informal food outlets are typified by *street food vending* that includes ready-to-eat foods or beverages prepared and/or sold in streets and public spaces by vendors or hawkers [15]. These vendors usually use portable booths, food carts, or trucks to sell food items [15]. Informal food outlets also include *mobile food outlets* that sell food out of a moveable vehicle, such as a truck, cart, trailer, kiosk or stand [16]. *Open-air markets* refer to those places with few or no permanent structures where buyers and sellers meet periodically and operate either daily or on a regular cycle [17]. Although farmer’s markets have been classified as formal markets by some researchers, [30] we included them in this literature review due to some similarities with the Latin informal food context; for example, some of these farmer’s markets have availability of ready-to-eat food. Farmer’s *markets* were defined as those that promote local and farm-fresh food [31].

### Search strategy

The systematic steps of the Cochrane Handbook for Systematic Reviews of interventions was used in this study [32]. PubMed, Web of Science, Scopus, and LILACS databases and manual scan of reference lists were used to identify potential articles. Articles were included if they reported instruments that measured the availability or type of healthy and unhealthy foods and beverages by informal food outlets. Instruments were included regardless of whether they assessed other aspects such as price, quality, variety, promotion, and placement, were written in English or Spanish, and published from January 1, 2010, to July 31, 2020. Articles were excluded if they: only employed qualitative methodology; reported opinions or attitudes; only measured marketing and advertising of food; reported on results of food environment interventions, only discussed food policies or food promotion; assessed foods sold only in formal stores (e.g., supermarkets, corner stores, grocery stores or convenience stores); measured availability of healthy/unhealthy products through physical distances (e.g., number of healthy/unhealthy products by shelf dimensions/space) or by GPS; or assessed food composition through bromatological analyses. Table 1 describes the databases used and searches terms.

**Table 1:** Databases and search terms

Database	Search terms
**PubMed**	1. “Nutritive value” AND “vendors” AND “measurement”. [MeSH Terms] OR [All Fields] 2. “Vendors” AND “food analysis” AND (surveys and questionnaires). [MeSH Terms] OR [All Fields] 3. “Commerce” AND “nutrition audits”. [MeSH Terms] OR [All Fields] 4. “Commerce” AND “nutritional characteristics”.[MeSH Terms] OR [All Fields] 5. “Nutrition audits” AND “food outlet”. [MeSH Terms] OR [All Fields] 6. “Food analysis” AND “retail food environment” AND (Surveys and questionnaires). [MeSH Terms] OR [All Fields] 7. “Street food” AND “nutrition values”. [MeSH Terms] OR [All Fields] 8. “Mobile food vendors” AND “Assessment”. [MeSH Terms] OR [All Fields]
**Web of Science**	1. (TS = (nutritive value AND vendors AND measurement)) AND DOCUMENT TYPES: (Article OR Abstract of Published Item) 2. (TS = (vendors AND food analysis AND surveys and questionnaires)) AND DOCUMENT TYPES: (Article OR Abstract of Published Item) 3. (TS = (commerce AND nutrition audits)) AND DOCUMENT TYPES: (Article OR Abstract of Published Item) 4. (TS = (commerce AND nutritional characteristics)) AND DOCUMENT TYPES: (Article OR Abstract of Published Item) 5. (TS = (nutrition audits AND food outlet)) AND DOCUMENT TYPES: (Article OR Abstract of Published Item) 6. (TS = (food analysis AND retail food environment AND surveys and questionnaires)) AND DOCUMENT TYPES: (Article OR Abstract of Published Item) 7. (TS = (street food AND nutritive value)) AND DOCUMENT TYPES: (Article OR Abstract of Published Item) 8. (TS = (Mobile food vendors AND Assessment)) AND DOCUMENT TYPES: (Article OR Abstract of Published Item)
**Scopus**	1. (TITLE-ABS-KEY (nutritive AND value) AND TITLE-ABS-KEY (vendors) AND TITLE-ABS-KEY (measurement)) 2. (TITLE-ABS-KEY (vendors) AND TITLE-ABS-KEY (food AND analysis) AND TITLE-ABS-KEY (surveys) AND TITLE-ABS-KEY (questionnaires)) 3. (TITLE-ABS-KEY (vendors) AND TITLE-ABS-KEY (food AND analysis) AND TITLE-ABS-KEY (surveys) OR TITLE-ABS-KEY (questionnaires)) 4. (TITLE-ABS-KEY (commerce) AND TITLE-ABS-KEY (nutrition AND audits)) 5. TITLE-ABS-KEY (commerce) AND TITLE-ABS-KEY (nutritional AND characteristics)) 6. (TITLE-ABS-KEY (nutrition AND audits) AND TITLE-ABS-KEY (food AND outlet)) 7. (TITLE-ABS-KEY (food AND analysis) AND TITLE-ABS-KEY (retail AND food AND environment) AND TITLE-ABS-KEY (surveys) OR TITLE-ABS-KEY (questionnaires)) 8. (TITLE-ABS-KEY (street AND food) AND TITLE-ABS-KEY (nutritive AND value)) 9. (TITLE-ABS-KEY (mobile AND food AND vendors) AND TITLE-ABS-KEY (assessment))
**LILACS**	1. (nutritive value and vendors) AND measurement 2. “Vendors” AND “food analysis” AND “surveys” AND “questionnaires” 3. “Commerce” AND “nutrition audits” AND (la:(“en”)) 4. “Commerce” AND “nutritional characteristics” AND (la:(“en” OR “es”))

#### Data collection process and synthesis of results

All citations were imported into an Excel spreadsheet and duplicates were removed manually. Two trained researchers reviewed and selected articles by title, abstract, and full text (JC and UL). Discrepancies were resolved by two independent researchers (CM and TA). For articles that met the inclusion criteria, data extraction was conducted by four researchers (JC, UL, TA, and CM). A data extraction form was developed and pilot tested on the first 10 selected articles and then refined. All researchers manually abstracted author, year, country, types of outlets, instrument description, healthy/unhealthy or produce/no produce or processed/without processing classification, and psychometrics tests into the extraction form. CM reviewed the information of each of the included articles. This information is presented descriptively in Table 2. Given the nature of our aim, a meta-analysis was not considered.

#### Quality of studies

The quality of published articles was evaluated using the (JBI) Critical appraisal checklist for analytical cross-sectional studies [53]. This checklist has eight questions that inquiries about inclusion criteria, subjects, and settings, validity and reliability of exposure measurements, standard criteria used for measurement of the condition, confounding factors, strategies to deal with confounding factors, validity and reliability of outcome measurements and appropriate statistical analysis [54]. Answers include yes, no, unclear, and not applicable. Overall appraisal encompasses “include”, “exclude”, and “seek further info”. This approach has been used elsewhere to assess the quality of studies [55]. Quality assessment was conducted by two independent reviewers (CM and UL) (Additional Material 1).

## Results

**Fig. 1:**
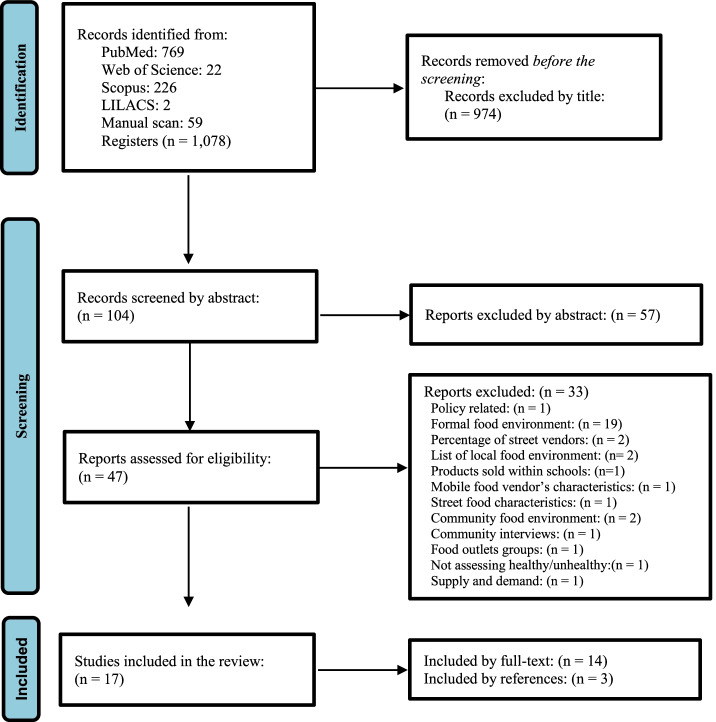
PRISMA Flow diagram: Identification and selection of studies

After removing duplicates, the literature search yielded 1078 articles, of which 47 were selected after being reviewed by title and abstract. This set included a total of 14 articles that were identified for a full review. In addition, 3 articles were selected through a manual search of the lists of references in the 14 included articles (Fig. 1).

In total, 17 studies described 13 instruments to classify how healthy/unhealthy are foods and/or beverages available within the informal food environment. The most common types of outlets considered in these reports were street food vendors, [38, 41, 42, 48, 50] farmers markets, [44, 45, 48, 49] open-air markets, [33–35], and mobile food vendors [33, 35, 36, 38, 41–43, 45, 46, 50, 52]. Tools included the Obesogenic Environment Study – observational tool for stores (ESAO-S), [33–35] different versions of the New Food Classification (NOVA), [36, 38, 39] adapted versions of Nutrition Environment Measures Survey – stores (NEMS-S), [43, 48] tools for farmers markets, [44, 49] standard Audit Forms for farmers markets, [50] the Food Retail Outlet Survey Tool (FROST), [45] assessment tool in US, [46] and audit tools from different countries [41, 42, 51, 52]. Six instruments were used in the Brazilian context [33–36, 39, 48]. In addition, an adaptation of the NEMS was used in the Mexican context (Mazatlán) [43]. Finally, NOVA classification was used in Mozambique [38, 41] and Tajikistan [42]. NOVA categorizes food and beverages according to food processing: unprocessed or minimally processed, processed culinary ingredients, processed foods and ultra-processed foods and drink products [36, 38, 39]. Further information on measurement tools is described in Table 2 (instrument description).

All the instruments evaluated the informal food environment through observation. All the tools that examined informal food outlets considered the availability and/or types of food and beverages. ESAO-S, [33–35], adaptation of NEMS [43, 48], FROST [45], and audit tools [51, 52] considered other characteristics such as variety, quality, quantity, price, advertising, promotion, and marketing. However, these variables were not used to classify food/beverages as healthy/unhealthy. Finally, instruments such as the adaptation of NEMS [48] and ESAO-S/HFSI [33, 34] created an overall indicator, considering several of the previous characteristics (e.g., variety, quality, price) to classify food/beverage outlets as healthy/unhealthy.

Some instruments included other topics such as tobacco, [45], and outlet characteristics (such as business name, type, and street address) [36, 38, 41, 42, 45, 46, 49, 50, 52]. Some tools measured the informal vending sites around schools [43], and bus stops [41]. Among the available instruments, several differences were found, including the way data was collected (e.g., checklists (availability yes/no), questionnaires).

Some instruments classified available and types of food and beverages as healthy and unhealthy [33–35, 43, 45, 46, 48–52] or produce (such as fresh products)/non-produce (such as processed food) [44]. Others used the NOVA classification based on food processing [36, 38, 39]. One instrument used fruit and beverages, homemade or industrial classification [41, 42]. Finally, some authors described the availability of food items as percentages or means, [35, 36, 41, 42, 44–46, 50–52] some others utilized calculated scores [33, 34, 48, 49]. The psychometric tests of the instruments included inter-rater reliability, [35, 39, 45, 48–50] test-retest reliability [35, 39, 45], and content, construct or face validity [35, 39, 45, 49]. Some others only performed pilot testing [44, 46, 48, 49, 51]. Seven studies did not report any psychometric tests [36, 38, 41, 42, 46, 51, 52]. In total, six studies reported psychometric tests either in the formal or informal food outlets, [35, 39, 45, 48–50] and one did so in terms of informal outlets only [49].

All the tools classified fruit and vegetables as healthy; however, some tools considered additional products within the healthy category including whole grains (e.g., bread and cereals), [45, 46, 48–50] plain/low-fat milk, [45, 48, 50, 51] nuts, [46, 48, 50] roots, and tubers, [48] beans, [48, 51] traditional dishes, [51] fresh meat [48, 49, 51] and fish [45, 51], eggs, [45, 48, 49, 51] reduced/low-fat yogurt, [48] some cheeses, [48, 49] and plain or mineral water [45, 50]. Studies based on NOVA classified unprocessed or minimally processed foods to be healthy, i.e., mainly of natural origin, preferably produced by agroecological methods, and appropriate and supportive of socially and environmentally sustainable food systems. These can include fresh fruits, fresh vegetables, fresh meat, milk, grains, legumes, nuts, teas, coffee, herb infusions, and tap and spring water in addition to fruit and vegetables [36, 38, 39]. A summary of these results is described in Table 2.

Almost all instruments classified processed foods/beverages [46, 48] as unhealthy, these included sweetened beverages, [33–35, 43, 49, 51, 52] corn or potato chips, [33–35] cream-filled cookies, [33–35] packed snacks (salty/fried, sweet, or frozen) [43, 52], non-whole-grain baked sweets, [44] savory items, [44] juice/ciders, [44] sugar-added items, [44] concentrated sweets, [44] refined sweets, [49] salty/fatty fare, [49] alcohol, [49, 51] cooking oils or fats, [51] jam, [51] hazelnut, [51] fried plantain, [51] processed meats, [51] pies, [51] cakes, [51] ice-cream, [51] chocolate, [51] pizza, [51] lasagna, [51] and ketchup [51]. And based on NOVA classification: *Unhealthy or ultra-processed food and drink products*: such as industrial formulations ready to be consumed, manufactured from five or even more ingredients commonly used in foods [39] (Table 2).

### Study quality

Based on the (JBI) Critical Appraisal Checklist for Analytical Cross-Sectional Studies, 9 out of 17 studies did not report if the exposure was measured validly and reliably [36, 38, 41–44, 46, 51, 52]. Due to the nature of the studies, almost all of them (*n* = 13) did not include confounding factors. The overall mean rating was “Included” (Additional Material 1).

**Table 2:** Measurement tools that evaluates “how healthy/unhealthy” are the food and beverages sold within the informal food outlets

Author, year	Country	Type of outlets	Instrument description	Healthy/unhealthy classification	Psychometric tests
Costa, et al., 2019 [33]	Belo Horizonte, Minas Gerais state, Brazil	Open-air food markets (e.g., fixed and mobile establishments).	Healthy food store index (HFSI): measures availability, variety, advertising of healthy items versus ultra-processed items. This index was based on an audit tool from ESAO study [34].	*Healthy* (fruits and vegetables) and *unhealthy* (sweetened beverages, corn chips, and cream-filled cookies). HFSI: classified outlets as healthy (positive score) or unhealthy (negative score). The score ranges from 1 to 16 [33]	Audit tool: inter-rater reliability ranges from 0.66 to 0.95. Test-retest ranges from 0.61 to 1 [35].
Duran et al., 2013 [34]	Sao Paulo, Brazil	Convenience stores, public-owned specialized fruit and vegetables (FV) markets, privately-owned specialized FV markets/stores, open-air food markets, corner stores, local grocery stores, large chain grocery stores, large chain supermarkets, delis.	Healthy food store index (HFSI): measures availability, variety and signage/promotion of the 10 most commonly purchased fruits and vegetables, and availability and signage/advertising of selected snacks items (sugar-sweetened beverages, chocolate sandwich cookies and processed corn chips) in the metropolitan area of Sao Paulo city. This index was derived from tools that assess healthy and unhealthy food availability, quality, variety, price, and signage/advertising or promotion.	*Healthy* (fruits and vegetables) and *unhealthy* (sweetened beverages, corn chips, and cream-filled cookies). HFSI: classified outlets as healthy (positive score) or unhealthy (negative score). The score ranges from 1 to 15 [34]	Tools: pilot tested. Inter-rater and test-retest reliability ranged from 0.50–0.95 [35].
Duran, et al., 2015 [35]	Sao Paulo, Brazil	Open-air-food markets (feiras-livres) (e.g., mobile or semi-fixed food markets)	Availability, variety, quality, pricing, signage and promotion of 10 most frequently purchased fruit and vegetables and the three most frequently consumed ultra-processed foods in Sao Paulo Metropolitan Region.	*Healthy* (fruit and salads: orange, banana, papaya, apple, tomato, onions, carrot, lettuce) or *unhealthy* (sugar-sweetened beverages: soda, sugar-free soda, sugar-sweetened nectar/juice, fruit-flavored drink mix, chocolate sandwich cookies and corn chips). Availability and quality are reported as percentage. Variety and price as a mean.	ESAO-S: test-retest reliability ranged from 0.61 to 1. Inter-rater reliability ranged from 0.66 to 0.95. For construct validity, these tools were able to discriminate between store types and different neighborhoods [35].
Leite, et al., 2012 [36]	Sao Paulo, Brazil	Fixed or mobile outlets.	Characteristics of stores, physical structure, inventory of food sold, which assessed the availability of food according to processing.	3 groups: *unprocessed or minimally processed food* (e.g., fresh meats and milk, grains, legumes, oilseeds, fruit and vegetables, roots and tubers, tea, coffee, herbal infusions and bottled water), *processed ingredients* (e.g., oils, fats, flour, pasta, starches and sugars, corn syrup, lactose and soy and milk protein), *ultra-processed food products* (e.g., bread, cereal bars, biscuits, chips, cakes, candies, ice cream and soda, frozen pasta and pizzas, sausages, breaded chicken, fish strips, canned or dehydrated soups, infant formulas and baby soups) [37].	Not available
Gelormini, et al., 2015 [38]	Maputo, Mozambique	Establishments selling ready-to-eat food or beverages for any venue on the streets, including carts, trucks, stands or any improvised informal setups.	Business’ operating hours and location, type of food products available, size of portions, price, and types of food packages. In addition to nutritional composition.		
Monteiro, et al., 2016 [39]	Brazil	Formal and informal environment.	According to food processing.	4 groups: *unprocessed or minimally processed foods* (e.g., fresh, squeezed, chilled, frozen, or dried fruits and leafy and root vegetables: grains such as brown, parboiled or white rice, corn cob or kernel, wheat berry or grain; legumes such as beans of all types, lentils, chickpeas; starchy roots and tubers such as potatoes and cassava, in bulk or package; fungi such as fresh or dried mushrooms; meat, poultry, fish and seafood, whole or in the form of steaks, fillets and other cuts, or chilled or frozen; eggs; milk, pasteurized or powdered; fresh or pasteurized fruit or vegetables juice without added sugar, sweeteners or flavors; grifts, flakes or flour made from corn, wheat, oats, or cassava; pasta, couscous and polenta made with flours, flakes or grits and water; tree and ground nuts and other oil seeds without added salt or sugar; spices such as pepper, cloves and cinnamon; and herbs such as thyme and mint, fresh or dried; plain yoghurt with no added sugar or artificial sweeteners added; tea, coffee, drinking water), *processed culinary ingredients* (e.g., salt mined or seawater; sugar and molasses obtained from cane or beet; honey extracted from combs and syrup from maple trees; vegetable oils crushed from olives or seeds; butter and lard obtained from milk and pork; and starches extracted from corn and other plants), *processed foods* (e.g., canned or bottled vegetables, fruit and legumes; salted or sugared nuts and seeds; salted cured or smoked meats; canned fish; fruit in syrup; cheeses and unpacked freshly made breads), *ultra-processed food and drink products* (e.g., casein, lactose, whey, and gluten, and some derived from further processing of food constituents, such as hydrogenated or interesterified oils, hydrolyzed proteins, soy protein isolate, maltodextrin, invert sugar and high fructose corn syrup, dyes and other colors, color stabilizers, flavors, flavor enhancers, non-sugar sweeteners, and processing aids such as carbonating, forming, bulking and anti-bulking, de-foaming, anti-caking and glazing agents, emulsifiers, sequestrants and humectants).	AUDITNOVA: Content validity index was 0.91 and inter-rater and test-retest reliability was > 0.80 [40].
Sousa, et al., 2019 [41] and Albuquerque, et al., 2019 [42]	Maputo, Mozambique Dushanbe, Tajikistan	Establishments selling ready-to-eat food or beverages for any venue on the streets, including carts, trucks, stands or any improvised informal setups.	Includes business characteristics, type of physical setup and mobility, gender of the vendor, operating hours, location, food availability, size of portions, prices and type of food packages.	Prevalence of three groups: 1) *fruits* (fleshy or dry), 2) *beverages* (e.g., soft drinks, water, fruit juices, milk, yogurt, alcoholic beverages, energetic drinks, homemade fermented beverages, tea and alcoholic drinks) and 3) *food other than fruit and beverages*. This group was classified as homemade or industrial (homemade – foods cooked and/or prepared at home, industrial – food industry products)	Not available
Bridle, et al., 2015 [43]	Mazatlán, Sinaloa, Mexico	Food outlets (restaurant, tortilleria, hot food cart, abarrotes)	Observational tools derived from a synthesis of literature review, consultation with local nutrition and policy practitioners, and NEM-S, NEMS-R, were used to evaluate food and beverages quantity, prices and promotions of informal or formal outlets around secondary schools. In outlets that offer ready to eat food or inside consumption, availability and prices of healthier options were collected through the menu.	Two groups of special interest were observed: 1) *healthy* (fruit and vegetables) and 2) *unhealthy* (beverages and packed snacks). Classifies outlets as more-healthier and less-healthier based on proportions. More healthier options were outlets with the widest variety and quantity of fresh fruit and vegetables. Less-healthier food outlet with a preponderance of packaged snacks and SSBs.	Not available
Lucan, et al., 2015 [44]	New York city, USA	Farmer’s markets	Evaluates all food items offered in farmer’s markets and fresh-produced items offered in nearby stores. In addition, this form inquiries about food quality (freshness and purity/naturalness), variety and price.	*Produce items* (fruit: fresh and dried varieties of generally-sweet, seed-bearing, whole produce, and vegetables: more-savory, seed-bearing, whole produce, shoots, leaves, flower buds, tubers, roots, bulbs, mushrooms, herbs) and two of non-produce items (other whole foods such as nuts, seeds, eggs, cheeses, and whole-grain products) and *refined or processed foods* (non-whole-grain baked sweets, savory items, juices/ciders, sugar-added items, concentrated sweets. Total food items sold at farmer’s markets were reported as proportions.	Pilot testing showed complete agreement between researchers for audit form items. Test-retest and validity were not reported [44].
Hosler, et al., 2011 [45]	New York city, USA	Permanent or mobile urban food stores and farmer’s market.	Contains two sections, the front section records availability, placement, prices of food and non-food items and stores’ physical characteristics. The back section contains information related to outside advertising and health promotion messages.	Availability of *healthier alternatives*, characterized by varieties that were low- or nonfat, higher-fiber, no-sugar added, or packed in plain water such as fruits: fresh, canned or frozen, vegetables: fresh dark green/orange, canned or frozen, grain and grain products: higher fiber bread (≥2 g/slice), brown rice, higher fiber pasta (≥5 g/2 oz), cereal (oatmeal), beans: dried or canned in water, plain water, milk (1% or skim), fish (tuna can in water, fresh fish filet, frozen fish filet), fresh eggs. Food items were reported as available (yes/no).	Inter-rater reliability for inside the store ranges from 0.59 to 1. Test-retest and inter-rater reliability for outside store ranges from 0.94 to 0.99 [45]. High degree of inter-rater and test-retest reliability implies construct validity [45].
Lucan, et al., 2013 [46]	New York city, USA	Vending vehicle (e.g. cart, stand or truck).	Direct observation regarding general characteristics, location, functionality of vending vehicles, type of vehicles, and location, types of food and beverages and interesting observations.	Types of vending: *healthier* (whole food like a fruit, vegetables, unprocessed grains, unsweetened nuts), *less-healthier* (processed and prepared food) and mixed (offering both). Vending items were classified as fresh produce, ethnic foods, other prepared foods, frozen novelty and others. Results were reported as numbers and percentages [47].	Pilot testing was conducted with essentially perfect agreement.
Martins, et al., 2013 [48]	Sao Paulo, Brazil	Supermarkets, grocery stores, convenience stores, farmer’s markets, produce markets, bakeries, butcheries, candy stores and street food stands.	NEMS-S: 5-minutes interview with the manager to collect: work days, time of opening and closure, the main products sold. Observation characteristics: physical space (fixed or mobile) and an inventory of the foods sold and a record of the availability of 33 food groups listed in the instrument, prices of foods and the quality of fruit and vegetables.	According to food processing and the Harvard Healthy Eating Pyramid recommendations: *Healthy* (fruit, non-starchy vegetables, roots and tubers except for potatoes and carrots, chicken, eggs, unprocessed seafood, milk, some cheeses, yogurts, brown rice, whole grains, beans, nuts), *intermediate* (oils, white rice, wheat flour, plain pasta, whole-grain bread) and *unhealthy* (mostly ultra-processed food) Healthy eating promotion score was used to classify food establishments according to food availability, price and quality.	Pilot-testing, internal consistency (0.71 for group 1, 0.068 for group 2, 0.93 for group 3), inter-rater reliability (0.61–0.80) and validity of mean score (*p* < 0.001).
Byker, et al., 2015 [49]	USA	Farmer’s markets	The tool has 27 unique items. Measures the availability and quality of food items and key characteristics about the operations of the market.	*Healthy* (fruit, vegetables, meats (pork: loin pastured, ground beef: lean ground beef with less than or equal to 10% fat, chicken: skinless chicken breast or whole chickens, fish: shellfish, whole fish, fish that has been sliced, eggs: regular and free range, cheeses: goats, cow’s milk, feta or lower calorie versions, and bread and grains: wheat, rye, other darker breads, samples: fruit and vegetables, salsa with chips). Food items were reported as available (yes/no).	Pilot testing inter-rater reliability (50–100% agreement), face validity.
Lucan, et al., 2020 [50]	New York city, USA	Storefront (e.g., convenience, supermarkets, groceries) and non-storefront (street food, mobile food outlet)	Identifies the name, type, street address and the hour of service of storefront and non-storefront food and beverages outlets.	*Healthy* (fruits, vegetables, nuts, whole grains, water and unflavored milk), *less-healthy* (refined sweets, salty/fatty fare, sugar sweetened beverages and alcohol), *neither healthy or unhealthy* (100% juice and diet drinks, eggs, cheese and poultry). Results were reported as percentage.	Inter-rater reliability was high.
Green, et al., 2020 [51]	Ghana and Kenya, Africa	Formal and informal outlets (e.g., kiosks, local vendors, vegetable/fruit stands/table tops).	Classifies food outlets as informal or formal. Informal outlets are those not movable, not permanent and/or those that have small-scale operation. In addition, items being sold and advertising are measured.	*Healthy* (e.g., raw/uncooked beef, pork, chicken fish/shellfish, milk, eggs, grains, cereals, roots, tubers, beans, peas, lentils, cashews, sesame seeds, jollof rice, banku, waakye, mango, watermelon, oranges, peppers, onions) or *unhealthy* (cooking oils or fats, jam, hazelnut spread, cola, soda, beer, vodka, wine, fried plantain, processed meats, pies, cake, ice cream, chocolate, pizza, lasagna, ketchup, shito) based on a nutrient profiling classification. Results are reported as a percentage of all records.	Pilot testing was performed in smaller areas.
Valdez, et al., 2012 [52]	South border Texas, USA	Mobile and home-based food vendors.	Includes demographics of vendors, characteristics of the business and work (length of time in business, initial investment, marketing area, percentage of household income from vending, hours of vending, food products sold by season, advertising, source of food products, scheduling strategy and target customers), working conditions and perceived relationships with customers.	*Healthier* (fruit and vegetables, juice with no added sugar or bottled water) and *less-healthy* (salty/fried, sweet or frozen snacks, sugar-sweetened beverages). Results are reported as percentage.	Not available

## Discussion

The purpose of this literature review was to evaluate available instruments that measure the healthfulness of products sold within the informal food outlets. In total, 17 articles were included and 13 measurement tools were identified. Most of the instruments were used in LMIC and all of them evaluated the food environment through observation. All the tools classified fruit and vegetables as healthy; however, some tools considered additional products within the healthy category. Furthermore, almost all instruments classified ultra-processed foods/beverages as unhealthy. Some instruments used other attributes such as variety, quantity, price, promotion, and advertising to generate a score that allows for classifying how healthy/unhealthy are food outlets. Six out of 13 instruments reported at least one psychometric test.

Studies from high-income countries have shown how inequality is associated with unhealthier food environments; i.e., people from lower-income neighborhoods have higher access to unhealthy products from formal food outlets such as convenience and grocery stores [56, 57]. However, most of this evidence came from the formal food environment. Conversely, a higher percentage of people from LMIC tend to buy products from informal food outlets such as street markets or street food stands [58]. In addition, evidence from LMICs shows that informal food outlets could contribute to > 10% of daily intake in adults and children [18]. The prevalence of food consumption away from home has increased around the world and within the LMICs [19–25]. Given the variety of products informal food outlets offer, evaluating the healthiness of these is a complex task. Thus, further research is needed to understand the contribution of these products to health outcomes.

Concerning the instruments per se, many differences make comparisons difficult. First of all, there are dissimilarities in the way instruments collect data, for instance, checklist (available yes/not) or questionnaires based on several elements including availability, accessibility, variety, quality, quantity, price, advertising, and/or promotion [43, 45]. Secondly, contrasts in foods/beverages considered healthy, i.e., only fruit and vegetables, or fruit and vegetables and whole grains, plain/low-fat milk, nuts, beans, traditional dishes, fresh meat and fish or eggs, reduced/low fat yogurt and plain or mineral water [45, 46, 48–51].

The accuracy of instruments assures that tools can be used by multiple researchers, at different times and can measure what is expected to be measured. Within this review, we found that only three tools reported inter-rater reliability, test-retest, and construct/content validity [35, 40, 45]. Nine studies reported at least one psychometric test, [35, 40, 44–46, 48–51] some others reported only pilot testing [44, 46, 48, 49, 51] or no testing [36, 38, 41–43, 52]. A systematic review that identified 48 tools to measure the food outlets reported that only 39% of them provided psychometric tests [59]. Given that food/beverages sold within the informal food outlets positively or negatively affect the dietary quality of children and adults, it is extremely important to have reliable and valid instruments to measure what is sold in these places.

There are some challenges when evaluating informal food outlets. This includes differences in the venues, for instance, open-air markets could be classified as informal food outlets in countries such as Brazil, [33] whereas, street vendors are the most common informal food outlet in other countries (Mozambique [38] and Mexico [43]). In addition, there are some elements of the informal food outlets that could hinder the assessment. Among these, are the abuse by the authorities of which they are victims in some contexts, their fear of being identified as not having business permits, and inconsistent hours of the points of sale. One of the most important relates to the itinerant or semi-itinerant vending such as vendors’ fear of being identified as not having business permits, and inconsistent schedules of outlets. Thus, future studies should consider these elements in the selection and/or creation of instruments to generate better quality information.

Although several instruments, mainly from high-income countries, have been used to measure formal food outlets, we found that 7 measurement tools were also used to measure informal food outlets. However, there are some limitations in evaluating a street food stand in the same way as a formal food store (such as a convenience store): 1) there are elements that cannot be present, at least to the same extent, in informal food outlets such as interior and exterior advertising or promotions, [45] 2) there are differences in how formal and informal outlets display food items, [60] and 3) there is proportionally higher availability of prepared food in informal outlets foods compared to formal food stores [58].

Available and/or newly developed tools should consider other aspects such as: 1) allow comparisons between countries at least in terms of the general characteristics of the environment, 2) include other elements such as price, degree of processing, hygiene, 3) include a local adaptation methodology before its implementation [61], 4) consider different contexts, and 5) allow comparisons between the formal and informal food environment. This last point is crucial because some formal food retailers such as “fondas” – family-run stalls or small canteens where food and drinks are sold in the Mexican context may offer products, in a quick-serve fashion and at a low cost, similar to what the informal food outlets could offer (such as in the case of food trucks).

Finally, given the increasing prevalence of NCDs, governments of all parts of the world have implemented a package of strategies including soda tax, the front of package labelling, and school policy regulations [62]. In countries such as Mexico, the food industry claims that products sold within informal food outlets could contribute to the high prevalence of overweight and obesity and not their products [27, 28]. However, this has not been substantiated by scientific research. Based on our results, further research is needed to: 1) characterize the informal food environment, 2) estimate the percentage of ultra-processed foods and basic non-industrialized foods in this environment, 3) understand the main contribution of these outlets to the diet and in the near future, if necessary, 4) to develop interventions to improve food environment aimed at promoting changes in offer and preparation by these outlets.

### Limitation and strengths

This study identified potential instruments that can be used to evaluate informal food outlets. We conducted a systematic literature review using four different databases; however, we did not search the “gray literature.” Therefore, we could have potentially missed some information. We used available definitions for informal food outlets; however, there may be alternative forms of informal food outlets in different regions of the world that we did not fully capture. Some studies were included despite not having a perfect quality assessment, so caution should be taken when these instruments are used to assess the healthfulness of informal food outlets.

## Conclusion

Although we found 13 instruments that have been developed or adapted to measure how healthy are food/beverages available at informal food outlets, only three performed inter-rater reliability, test-retest reliability, and validity, of which they were not used to exclusively measure informal food outlets. There are many drawbacks to measuring informal food outlets in the same way as formal food outlets. Therefore, it is necessary to develop an instrument that measures the elements specific to the informal food environment.

Additional research in this area is urgently needed to better understand a key aspect of the food environment that may be a central contributor to poor diets that are increasingly associated with the obesity and Non-communicable disease (NCD) pandemic.

### Registration and protocol

There is no available registration number. This protocol was reviewed and approved by the Ethical Board of the National Institute of Public Health Mexico (Number: CI 1684). This systematic literature review followed PRISMA guidelines (Additional Material 2) [63].

## Supplementary Information

**Additional file 1: Additional Material 1**. JBI Critical appraisal checklist for analytical cross-sectional studies

**Additional file 2.** PRISMA 2020 Checklist.

## Data Availability

All data generated or analyzed during this study are included in this published article.

## Abbreviations

FROST: Food Retail Outlet Survey Tool
FV: Fruit and vegetables
GPS: Global Positioning System
HFSI: Healthy food store index
LMIC: Low- and middle-income countries
NOVA: New Food Classification
NCDs: Non-communicable diseases
NEMS-S: Nutrition Environment Measures Survey – stores
NEMS-R: Nutrition Environment Measures Survey – restaurants
ESAO-S: Obesogenic Environment Study – observational tool for stores
USA: United States of America
