# Supplementary material for: Systematic literature review of instruments that measure the healthfulness of food and beverages sold in informal food outlets
Source: Int J Behav Nutr Phys Act. 2022 Jul 16;19:89. doi: 10.1186/s12966-022-01320-1 (PMC9288710; doi:10.1186/s12966-022-01320-1)
Supplement: Supplementary file 1 — Additional file 1: Additional Material 1. JBI Critical appraisal checklist for analytical cross-sectional studies [file 12966_2022_1320_MOESM1_ESM.docx]

**Additional Material 1. JBI Critical appraisal checklist for analytical cross-sectional studies**

| **Author, year** | **Q1** | **Q2** | **Q3** | **Q4** | **Q5** | **Q6** | **Q7** | **Q8** | **Overall appraisal** |
| --- | --- | --- | --- | --- | --- | --- | --- | --- | --- |
| Costa, et al., 2019 (1) | Y | Y | Y | Y | Y | Y | Y | Y | Include |
| Duran et. al., 2013(2) | Y | Y | Y | Y | Y | Y | Y | Y | Include |
| Leite, et. al., 2012(3) | Y | Y | N | Y | NA | NA | Y | Y | Include |
| Gelormini, et. al., 2015(4) | Y | Y | N | Y | NA | NA | Y | U | Include |
| Monteiro, et. al., 2016(5) | NA | NA | Y | NA | NA | NA | Y | NA | Include |
| Sousa, et al., 2019(6) | Y | Y | N | Y | NA | NA | Y | Y | Include |
| Albuquerque, et. al., 2019(7) | Y | Y | N | Y | Y | Y | Y | Y | Include |
| Bridle, et al., 2015 (8) | Y | Y | N | Y | NA | NA | U | Y | Include |
| Lucan, et al., 2015 (9) | Y | Y | N | Y | Y | Y | Y | Y | Include |
| Hosler, et al., 2011(10) | Y | Y | Y | Y | NA | NA | Y | Y | Include |
| Duran, et al., 2015 (11) | Y | Y | Y | Y | NA | NA | Y | Y | Include |
| Lucan, et al., 2013 (12) | Y | Y | N | Y | NA | NA | Y | Y | Include |
| Martins, et. al., 2013(13) | Y | Y | Y | Y | NA | NA | Y | Y | Include |
| Byker, et. al., 2015(14) | Y | Y | Y | Y | NA | NA | Y | Y | Include |
| Lucan, et al., 2020 (15) | Y | Y | U | Y | NA | NA | Y | Y | Include |
| Green, et al., 2020 (16) | Y | Y | N | Y | NA | NA | Y | Y | Include |
| Valdez, et al., 2012 (17) | Y | Y | N | Y | NA | NA | U | Y | Include |
| Y- yes, N- no, U- unclear, NA- not applicable | | | | | | | | | |

**References**

1. Costa BVL, Menezes MC, Oliveira CDL, Mingoti SA, Jaime PC, Caiaffa WT, et al. Does access to healthy food vary according to socioeconomic status and to food store type? an ecologic study. BMC Public Health. 2019;19(1):775.

2. Duran AC, Diez Roux AV, Latorre Mdo R, Jaime PC. Neighborhood socioeconomic characteristics and differences in the availability of healthy food stores and restaurants in Sao Paulo, Brazil. Health Place. 2013;23:39-47.

3. Leite FH, Oliveira MA, Cremm EC, Abreu DS, Maron LR, Martins PA. Availability of processed foods in the perimeter of public schools in urban areas. J Pediatr (Rio J). 2012;88(4):328-34.

4. Gelormini M, Damasceno A, Lopes SA, Malo S, Chongole C, Muholove P, et al. Street Food Environment in Maputo (STOOD Map): a Cross-Sectional Study in Mozambique. JMIR Res Protoc. 2015;4(3):e98.

5. Monteiro C, Cannon G, Levy R, Moubarac J-C, Jaime P, Martins AP. NOVA: the star shines bright. World Nutrition. 2016;7:28-38.

6. Sousa S, Gelormini M, Damasceno A, Lopes SA, Malo S, Chongole C, et al. Street food in Maputo, Mozambique: Availability and nutritional value of homemade foods. Nutr Health. 2019;25(1):37-46.

7. Albuquerque G, Morais I, Gelormini M, Casal S, Damasceno A, Pinho O, et al. Street food in Dushanbe, Tajikistan: availability and nutritional value. Br J Nutr. 2019;122(9):1052-61.

8. Bridle-Fitzpatrick S. Food deserts or food swamps?: A mixed-methods study of local food environments in a Mexican city. Soc Sci Med. 2015;142:202-13.

9. Lucan SC, Maroko AR, Sanon O, Frias R, Schechter CB. Urban farmers' markets: accessibility, offerings, and produce variety, quality, and price compared to nearby stores. Appetite. 2015;90:23-30.

10. Hosler AS, Dharssi A. Reliability of a survey tool for measuring consumer nutrition environment in urban food stores. J Public Health Manag Pract. 2011;17(5):E1-8.

11. Duran AC, Lock K, Latorre Mdo R, Jaime PC. Evaluating the use of in-store measures in retail food stores and restaurants in Brazil. Rev Saude Publica. 2015;49.

12. Lucan SC, Varona M, Maroko AR, Bumol J, Torrens L, Wylie-Rosett J. Assessing mobile food vendors (a.k.a. street food vendors)--methods, challenges, and lessons learned for future food-environment research. Public Health. 2013;127(8):766-76.

13. Martins PA, Cremm EC, Leite FH, Maron LR, Scagliusi FB, Oliveira MA. Validation of an adapted version of the nutrition environment measurement tool for stores (NEMS-S) in an urban area of Brazil. J Nutr Educ Behav. 2013;45(6):785-92.

14. Byker Shanks C, Jilcott Pitts S, Gustafson A. Development and Validation of a Farmers' Market Audit Tool in Rural and Urban Communities. Health Promot Pract. 2015;16(6):859-66.

15. Lucan SC, Maroko AR, Patel AN, Gjonbalaj I, Elbel B, Schechter CB. Healthful and less-healthful foods and drinks from storefront and non-storefront businesses: implications for 'food deserts', 'food swamps' and food-source disparities. Public Health Nutr. 2020;23(8):1428-39.

16. Green MA, Pradeilles R, Laar A, Osei-Kwasi H, Bricas N, Coleman N, et al. Investigating foods and beverages sold and advertised in deprived urban neighbourhoods in Ghana and Kenya: a cross-sectional study. BMJ Open. 2020;10(6):e035680.

17. Valdez Z, Dean WR, Sharkey JR. Mobile and home-based vendors' contributions to the retail food environment in rural South Texas Mexican-origin settlements. Appetite. 2012;59(2):212-7.
